# Heteromeric Kv7.2 current changes caused by loss-of-function of *KCNQ2* mutations are correlated with long-term neurodevelopmental outcomes

**DOI:** 10.1038/s41598-020-70212-w

**Published:** 2020-08-07

**Authors:** Inn-Chi Lee, Jiann-Jou Yang, Swee-Hee Wong, Ying-Ming Liou, Shuan-Yow Li

**Affiliations:** 1 Division of Pediatric Neurology, Department of Pediatrics, Chung Shan Medical University Hospital, Taichung, Taiwan; 2 Institute of Medicine, School of Medicine, Chung Shan Medical University, #110, Section 1, Chien-Kuo North Road, Taichung, 402 Taiwan; 3 Genetics Laboratory, Department of Biomedical Sciences, Chung Shan Medical University, Taichung, Taiwan; 4 Department of Life Sciences, National Chung-Hsing University, Taichung, Taiwan; 5 The iEGG and Animal Biotechnology Center, Rong Hsing Research Center for Translational Medicine, National Chung Hsing University, Taichung, 40227 Taiwan

**Keywords:** Neuroscience, Medical research, Neurology, Pathogenesis

## Abstract

Pediatric epilepsy caused by *KCNQ2* mutations can manifest benign familial neonatal convulsions (BFNC) to neonatal-onset epileptic encephalopathy (EE). Patients might manifest mild to profound neurodevelopmental disabilities. We analysed c.853C > A (P285T) and three mutations that cause KCNQ2 protein changes in the 247 position: c.740C > T (S247L), c.740C > A (S247X), and c.740C > G (S247W). S247L, S247W, and P285T cause neonatal-onset EE and poor neurodevelopmental outcomes; S247X cause BFNC and normal outcome. We investigated the phenotypes correlated with human embryonic kidney 293 (HEK293) cell functional current changes. More cell-current changes and a worse conductance curve were present in the homomeric transfected S247X than in S247L, S247W, and P285T*.* But in the heteromeric channel, S247L, S247W and P285T had more current impairments than did S247X. The protein expressions of S247X were nonfunctional. The outcomes were most severe in S247L and S247W, and severity was correlated with heteromeric current. Current changes were more significant in cells with homomeric S247X, but currents were “rescued” after heteromeric transfection of *KCNQ2* and *KCNQ*3. This was not the case in cells with S247L, S247W. Our findings support that homomeric current changes are common in *KCNQ2* neonatal-onset EE and *KCNQ2* BFNC; however, heteromeric functional current changes are correlated with long-term neurodevelopmental outcomes.

## Introduction

*KCNQ2* (OMIM 602235)-associated seizures usually occur during the first week after birth and can contribute to benign familial neonatal convulsions (BFNC), benign familial neonatal-infantile seizures (BFNIS), benign familial infantile seizures (BFIS)^1–5^, and neonatal-onset epileptic encephalopathy (EE)^6–8^. Mutations in *KCNQ2*, a voltage-gated potassium channel gene at 20q13, are usually inherited in an autosomal-dominant manner in benign epileptic syndromes^1,2^. Patients with BFNC usually have seizures with a predicted benign course and predicted good neurodevelopmental outcomes^1–3,9,10^. On follow-up, about 30% of patients with inherited *KCNQ2* mutations might have recurrent seizures beyond neonatal age^10^. Most neonatal-onset EE, mutations are de novo, and patients present with severe seizures and grave neurological consequences. Seizures often remit as the patients become older, but the patients usually have intellectual developmental delays or autism^11,12^. At present, however, outcomes cannot be accurately predicted.

Functional KCNQ channels are homo- or heteromers of four subunits each containing 6 transmembrane domains (S1–S6), which include a voltage sensor in S1–S4 and S5–S6, and a loop between S5–S6 that builds the ion channel pore, a cytoplasmic N-terminal, and a long C-terminal region with complex functions exhibiting interactions between syntaxin, phosphatidylinositol 4,5-bisphosphate, ankyrin-G, Syn-1A, and A-kinase anchoring protein^2,5,13–18^. The important mechanism governing the functional expression of *KCNQ2* includes: first, the networks of interactions between the pore helix and the selectivity filter, and between the pore helix and the S6 domain that are responsible for Kv7.2 current; and second, controlling KCNQ2 protein to the plasma membrane by the distal part of the C-terminus for channel trafficking and assembly and the proximal half of the C-terminus for channel modulation by interacting with calmodulin (CaM)^19,20^. The C-terminal tail contains two helical domains (helices A and B): helix A contains the consensus CaM-binding IQ motif, and helix B mediates Ca2^+^-dependent CaM binding^21,22^. CaM interacts with CaM molecules and maintains stability in the potassium channel. Mutations in *KCNQ2* of the C-terminus affect CaM binding and functional modulation^23^. KCNQ2 protein is widely expressed in the hippocampus, neocortex, and cerebellar cortex of the human brain and is encoded for voltage-gated potassium channel subunits that underlie the M-current, a repolarizing current that limits repetitive firing during long-lasting depolarizing inputs^4,24,25^. In the *KCNQ2* gene, mutations can cause a haploinsufficiency, and a more severe dominant-negative effect by a loss-of-function^26–29^. Loss-of-function accounts for the majority of *KCNQ2*-induced neonatal-onset EE^26–29^, but gain-of-function, which is presumed to be the mechanisms in several mutations^30–33^.

The different phenotypes and neurodevelopmental outcomes of *KCNQ2* mutations might be determined by the degree of functional disability of the mutations. Other probable factors include parental germline mosaicism^7,8^, genetic modifiers^14,34^, environmental factors, and when seizures ceased^35^. The phenotype determined by in vitro functional current changes is consistent with Miceli et al.^36^, who reported the differences between R213Q and R213W, which showed that R213Q leads to a significantly greater functional change than does R213W, and that it yields distinct phenotypical outcomes and neurodevelopmental outcomes: R213W caused BFNC and R213Q caused neonatal-onset EE.

One study^29^ reported the role of the A294V mutation in the S6 of KCNQ2 protein-related neonatal-onset EE and the role of the A294G mutation-related BFNCs. Both mutations result in loss-of-function effects, and reduced currents mediate homomeric and heteromeric channels. The A294V mutation specifically affects the targeting of the channel to the initial axon segment, whereas the A294G mutation does not. This finding was related to the different phenotypes in the two mutations.

The phenotypes and genotypes of *KCNQ2*-associated epilepsy continue to be investigated, and some case series^6–8,10,23,37^ and functional studies have been conducted^26–33,38,39^. The precise genotype–phenotype correlation in *KCNQ2*-related epilepsy is not fully understood. Studies have claimed that homomeric current change is common in *KCNQ2* neonatal-onset EE^28,29,36,40^. We hypothesized that homomeric current change by variants is correlated with neurodevelopmental outcomes. We investigated various *KCNQ2* variants in the same position as amino acids that lead to neonatal-onset EE and BFNCs to analyse functional current changes in HEK293 cells.

## Methods and materials

### Participants

**Figure 1:**
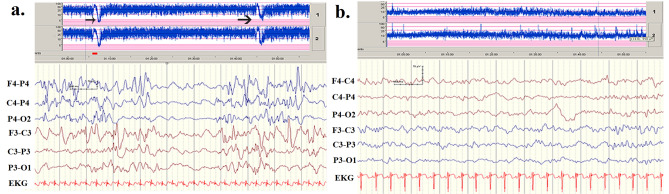
(**a**) The patient with a G285T mutation experienced seizures on neonatal day 2. His seizures did not remit after treatment with numerous antiepileptic drugs. Automated and conventional EEG monitoring revealed two seizures within 1 h (black arrows). His interictal EEG depicted a burst-suppression pattern. (**b**) The patient with the R448X mutation had the BFNC phenotype. He had his first seizure the age of 2 days. His seizures were controlled with intravenous phenobarbital and phenytoin. The EEG indicated no remarkable findings.

Three infants in our clinic carrying de novo c.740C > T (S247L), de novo c.853 C > A (P285T), and familial c.1342C > T (R448X) mutations presented with neonatal-onset seizures. Patients with S247L and P285T exhibited neonatal-onset EE, burst-suppression in electroencephalogram (EEG) recordings (Fig. 1a), and frequent neonatal seizures^37^. Patients with R448X experienced neonatal seizures with the BFNC phenotype (Fig. 1b). Parents gave written informed consent for their child to participate.

To investigate the phenotypes correlated with in vitro HEK293 cell functional cell-current change and KCNQ2 protein expression on cell membranes, we analysed three variants that cause different amino acid changes at KCNQ2 protein position 247: c.740C > A (S247X), c.740C > T (S247L), and c.740C > G (S247W). The patient with S247W had poor neurodevelopmental outcome and was not able to sit without support at 2 years and 5 months old^41^. S247W^41^, S247L and P285T cause neonatal-onset EE and poor neurodevelopmental outcomes; S247X^42^ causes BFNCs with relatively more favourable neurodevelopmental outcomes. We reported P285T^37^ as a novel mutation. In this study, we investigate the in vitro functional cell-current change of P285T. The control database includes the Exome Aggregation Consortium (Exac) (https://exac.broadinstitute.org) and Taiwan BioBank (https://taiwanview.twbiobank.org.tw), which contains the frequencies of variants in the general population. Sequencing data were compared with the GenBank reference sequences and version numbers of *KCNQ2* genes (NM_172107.3). We transfected these variants into HEK293 cells and then used immunostaining and western blotting to investigate KCNQ2 protein expression on HEK293 cell membranes.

### Transfecting variants to HEK293 cells

HEK293 cell cultures were maintained at 37 °C in a humidified 5% CO_2_ incubator. The vectors, pLEGFP and pTaqRFP, which contain the DNA fragments encoding wild-type and mutant *KCNQ2*, were transfected to HEK293 cells using a reagent (lipofectamine; Thermo Fisher Scientific: Invitrogen, USA). *KCNQ2* mutations were created using a kit (QuickChange; Stratagene, La Jolla, CA, USA) and verified using sequencing.

### Protein separation in cytoplasm and on cell membranes

HEK293 cells (6 × 10^6^/well) were seeded in 3 wells in a 10-cm cell culture dish. The cells were washed two or three times with phosphate buffered saline (PBS) that contained 4 g of NaCl, 0.1 g of KCl, 0.72 g of Na_2_HPO_4_, and 0.13 g of KH_2_PO_4_, and had an adjusted pH of 7.4. They were then added to cells that contained sucrose in a homogeneous solution (40 mM of Tris–HCl [pH 7.4], 0.34 M of sucrose, 10 mM of ethylenediamine tetraacetic acid [EDTA], 1 mM of MgSO_4_) before they were used, and they were then added to 1 mL of 1-mM phenylmethyl sulfonyl fluoride (PMSF). The cell mixture was placed on ice, sonicated 3 times for 2 min each time (intensity: 30), and then slowly added to a centrifuge tube that contained 40 mM of Tris–HCl [pH 7.4], 50% sucrose, 10 mM of EDTA, 1 mM of MgSO_4_, and 2 mM of NaN_4_. The mixture was then added to a solution (0.75 mL) with 40 mM of Tris–HCl [pH 7.4], 20% sucrose, 10 mM of EDTA, 1 mM of MgSO_4_, 2 mM of NaN_4_, and complete shock cell homogenates. The solution was then centrifuged in an ultra-high-speed rotor (55-Ti; Beckman Coulter Taiwan, Taipei) at 4 °C and 26,200 rpm for 90 min. After the solution had been centrifuged, the cellular proteins rose to the top of the liquid^43^.

### Western blotting

Samples were diluted to at least 1:5 with sample buffer, heated at 95 °C for 5 min, and then stored at 4 °C until they were used. The gel was run at 80 V for 10 min and then at 130 V for 3 h. Polyvinylidene difluoride (PVDF) membranes (Merck Millipore, Burlington, MA, USA) were soaked in methanol for 1 min and then placed in the “sandwich” chamber with 2 fiber pads and 2 filter papers, all soaking in old transfer buffer. The “sandwich” was transferred for 1.5 h at 100 V at 4 °C. The membranes were then shaken in 5% nonfat dry milk in PBS for 1 h on a shaker at room temperature; they were then incubated with a primary anti-KCNQ2 antibody (1:200) (Thermo Fisher, Waltham, MA, USA) in 1% milk at 4 °C on a shaker overnight. The next day, after it had been washed with PBST (phosphate buffer saline + Tween 20) 4 times for 10 min each time, the membrane was incubated with a secondary antibody (anti-rabbit) (1:3,000) (Gentex, Carbondale, PA, USA) in 1% milk prepared with PBS for ~ 1 h at room temperature, rinsed with PBST 4 times for 10 min each time, and then analysed using a western blotting detection kit (Advansta, Menlo Park, CA, USA). Anti-glyceraldehyde phosphate dehydrogenase (GAPDH) antibody was used as a loading control.

### In vitro functional study

#### Expression in HEK293 cells, and whole-cell patch-clamp analysis

HEK293 cells were maintained in Dulbecco’s modified Eagle’s medium (DMEM) (Biowhittaker, Walkersville, MD, USA) supplemented with 10% fetal bovine serum (FBS), penicillin (100 U/ml), streptomycin (100 U/ml), and 2 mM l-glutamine (Lonza, Walkersville, MD, USA). *KCNQ2* mutations were created using a kit (QuickChange; Stratagene, La Jolla, CA, USA) and verified using sequencing^39^.

#### Whole-cell patch-clamp analysis

For electrophysiological analysis, the cells were bathed in modified Tyrode’s solution (125 mM NaCl, 5.4 mM KCl, 1.8 mM CaCl_2_, 1 mM MgCl_2_, 6 mM glucose, and 6 mM HEPES [pH 7.4]). Patch-pipettes had a resistance of 3–4 Ω when filled with pipette solution (125 mM of potassium gluconate, 10 mM of KCl, 5 mM of HEPES, 5 mM of ethylene glycol tetraacetic acid [EGTA], 2 mM of MgCl_2_, 0.6 mM of CaCl_2_, and 4 mM of adenosine 5´-triphosphate disodium salt hydrate [Na_2_ATP] [pH 7.2]).

To measure the voltage dependence of activation, the cells were clamped using 3-s conditioning voltage pulses to potentials between − 80 mV and + 40 mV in 10-mV increments from a holding potential of − 80 mV. Data acquisition and analysis were done using electrophysiology data acquisition and analysis software (Clampex 10.0; Molecular Devices, Sunnyvale, CA, USA). The data were then fit to a Boltzmann distribution of the following form: *G*/*Gmax* = 1/(1 + *exp*[(*V* − *V½*)/*dx*]). Cell capacitance was obtained by reading the settings for the whole-cell input capacitance neutralization directly from the amplifier^44^. *KCNQ2* mutations variants and wild-type (WT) were transfected into HEK293 cells to investigate the functional changes that cause cell-current changes^36,45^.

### Ethics committee approval

Ethical approval of the study was provided by Chung Shan Medical University Hospital’s Internal Review Board (IRB #: CS13036). Informed consents were obtained from parents of patients. All experiments were performed in accordance with relevant named guidelines and regulations.

### Statistical analysis

Data are mean ± standard deviation (SD). Significant differences were evaluated using an independent *t* test or an analysis of variance (ANOVA) test. Significance was set at *p* < 0.05.

## Results

### Clinical presentations and neurodevelopmental outcomes in various KCNQ2 mutations

The clinical presentations in patients with S247X, S247L, S247W, and P285T are summarized in Table 1. Neurodevelopmental outcome was more favourable in the patient carrying the S247X mutation, who presented with BFNC, than in those carrying the S247L, S247W, and P285T mutations, who presented with neonatal-onset EE identified based on burst suppression in EEG recordings (Table 1). The patient with S247L had a severe cognitive disability without any language development and could not walk at the age of 3 years despite partial remission of seizures at the age of 4 months but with recurrent febrile seizures. The patient with P285T had frequent neonatal seizures and apnoea. Her seizures became less frequent after the age of 2 months, but she had a severe cognitive disability at the age of 3 years. The neurodevelopmental outcomes were poor in three cases of *KCNQ2*-associated neonatal-onset EE. The patients could not sit without support and were without language development at the age of 2 years (Table 1).

**Table 1:** Clinical presentations in patients with the four mutations.

Genotype in patients	c.740C > A (S247X)^42^	c.740C > G (S247W)^41^	c.740C > T (S247L)	c.853 C > A (P285T)
Pattern of inheritance	Inherited, autosomal dominance	De novo	De novo	De novo
Functional domain	S5	S5	S5	Pore domain
Family history	+ (5 affected family members)	No	No	No
First seizure day	Day 5	Day 3	Day 3	Day 2
Seizure frequency before drug control	+	+++	+++	+++
Antiepileptic drugs	PB	Intravenous PB, PHT, oral pyridoxine, PB, and SAB	Intravenous PB, PHT then oral PB, SAB, CLN	Intravenous PB, PHT then oral PB, SAB, CLN, OXC
Age when seizure-free	6 months	No remission of seizures	Partial remission of seizures at 4 months, with recurrent febrile seizures	Partial remission of seizures at 2 months
Seizure types	Bicycling of legs and arms, apnea	Multifocal with left or right head deviation and upper and lower limb involvement	Generalized tonic	Generalized tonic
Initial EEG	Central sharp waves or spikes	Burst-suppression pattern	Burst-suppression pattern	Burst-suppression pattern
MRI/ CT	Normal	Normal MRI at 18 days and normal third CT scan at 30 days	Basal ganglion	Thin corpus callosum
Neurodevelopmental outcomes	Unremarkable	Poor at 2 year and 5 months: head control and social smiling but inability to sit without support, muscle hypotonia, dystonic features	Poor at 3 years: inability to sit without support, inability to walk, lack of language production, severe cognitive disability	Poor at 2 years and 4 months: inability to sit without support, inability to walk, lack of language production, severe cognitive disability

PHT, phenytoin; OXC, oxcarbazepine; TOP, topiramate; PB, phenobarbital; SAB, vigabatrin; CLN, clonazepam; MRI, magnetic resonance imaging; CT, computed tomography; EEG, electroencephalography; ++ +, daily; ++, weekly; +, less than weekly; ADHD, attention deficit and hyperactivity; Dev. Del./Int. Dis., Developmental delay/intellectual disability. The sequence data of each patient were checked against the GenBank reference sequence and version number of *KCNQ2* gene (NM_172107.3).

**Figure 2:**
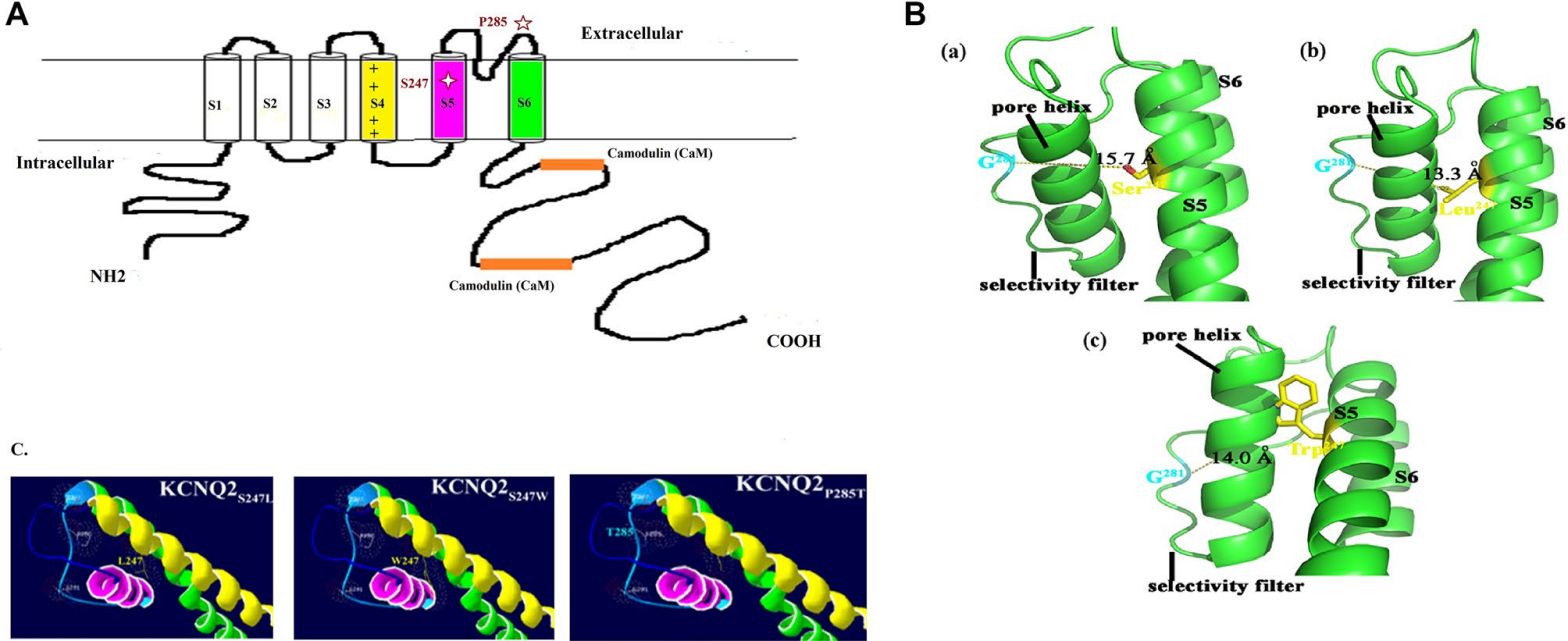
(**A**) The schematic representation of KCNQ2 subunit with the position of the mutations of S247L, S247W and P285T. (**B**) Molecular modelling of KCNQ2 channel proteins (NM_004518) was generated by using the Phyre2 tool (Protein Homology/analogY Recognition Engine V 2.0) with the NP_004509.2 protein sequence. This tool is available on a website for protein modelling, prediction, and analysis based on the CryoEM structure of the Xenopus KCNQ1 channel^54^. The predicted 3D model of the KCNQ2 channel protein (c5vmsA_.1.pdb) was then used to analyse the structural difference between WT and mutant cells by using Swiss-PDBV (4.1.0 https://spdbv.vital-it.ch/) and PyMOL (https://www.pymol.org/), respectively. (**a**) WT S247, (**b**) S247L mutation, and (**c**) S247W mutation. The distance between residue 281 (G) on the selectivity filter and residue 247 on S5 of KCNQ2 was estimated as 15.7 Å for the WT (S247), 13.3 Å for the S247L mutant, and 14 Å for S247W mutant. Ion accessibility through the channel pore decreased for both mutants (S247L and S247W) at the S5 segment with bulkier side chain replacements compared with the WT (S247). (**C**) Mutation sites at the selectivity filter (P285T) might alter accessibility for potassium ions through the channels. Yellow color indicates S4; pink, S5; green, S6.

Figure 2 is a schematic of the KCNQ2 subunit showing the positions of the S247L, S247W, and P285T mutations. The S247 protein is located in S5, and the P285 protein is in the selectivity filter of the pore domain (Fig. 2A). The distance between residue 281 (G) on the selectivity filter and residue 247 on S5 of KCNQ2 was estimated as 15.7 Å for the WT (S247), 13.3 Å for the S247L mutant, and 14 Å for the S247W mutant. Ion accessibility through the channel pore decreased for both mutants (S247L and S247W) at the S5 segment, with bulkier side chain replacements compared with the WT (S247) (Fig. 2B, C).

### Electrophysiological properties of S247L, S247W, and S247X mutations in KCNQ2

**Figure 3:**
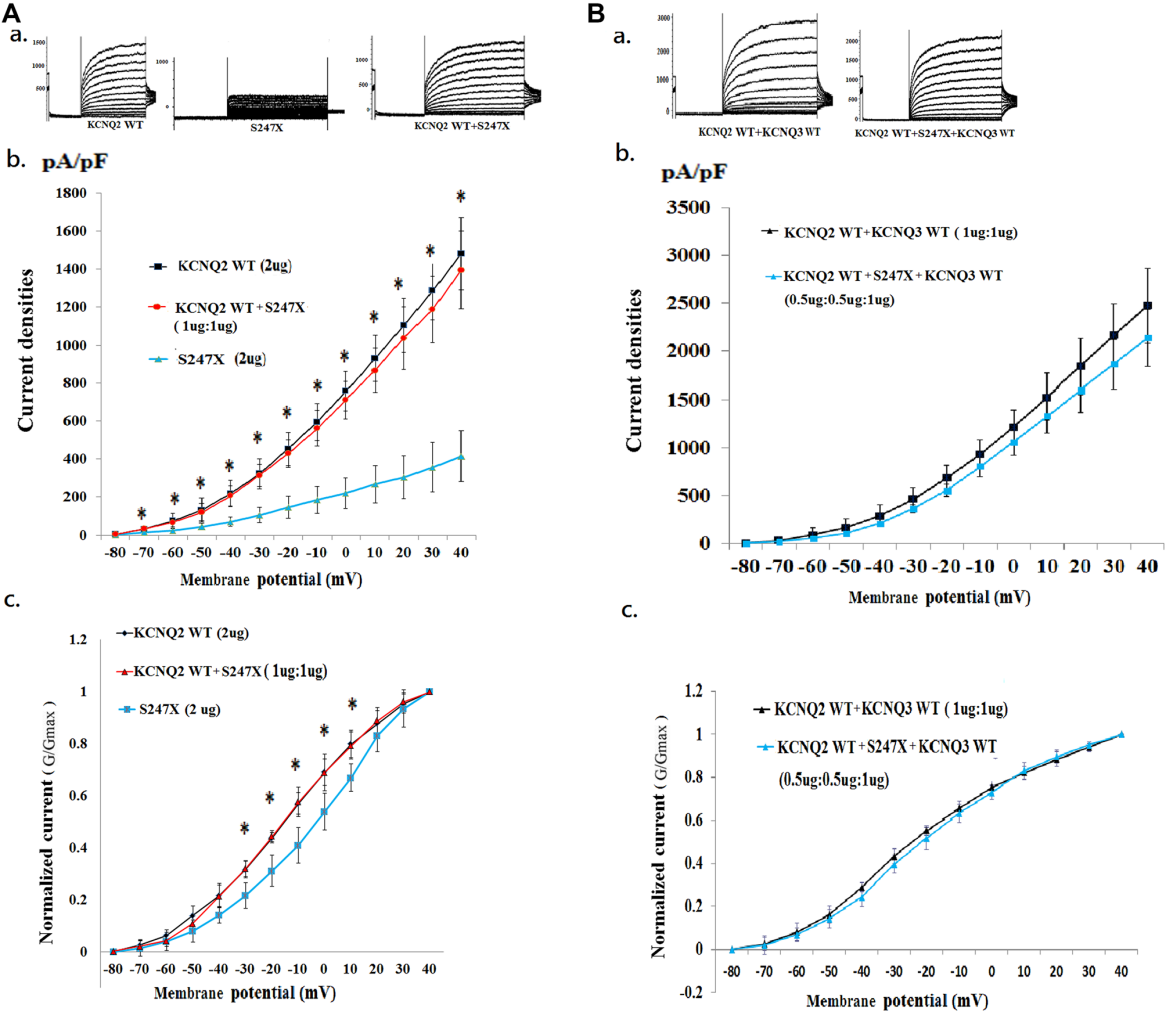
Analysis of electrophysiological properties of HEK293 cells in KCNQ2 homomeric and heteromeric S247X channels. (**A**) (**a**) Representative current traces of *KCNQ2* WT (2 μg) (n = 20), S247X (2 μg) (n = 10), and *KCNQ2* WT + S247X (1 μg:1 μg) (n = 10). (**b**) Current density versus membrane potential (from − 80 to 40 mV) for *KCNQ2* WT, S247X, and *KCNQ2* WT + S247X. The current density demonstrated the lowest conductance–current curve in homomeric S247X for each membranous potential (− 80 to 40 mV), but the curve corresponds more closely with the *KCNQ2* WT cells after the addition of *KCNQ2.* *p < 0.05 for *KCNQ2* WT versus S247X. (**c**) Normalized currents (G/G_max_) versus voltage (from − 80 to 40 mV) for *KCNQ2* WT (2 μg), S247X (2 μg), and *KCNQ2* WT + S247X (1 μg:1 μg). The cells transfected with homomeric S247X exhibited lower normalized currents (from − 30 to 10 mV) than did cells with *KCNQ2* WT. *p < 0.05 for *KCNQ2* WT versus S247X. (**B**) (**a**) Representative current traces of *KCNQ*2 WT + *KCNQ3* WT (1 μg + 1 μg) (n = 10) and *KCNQ2* WT + S247X + *KCNQ*3 (0.5 μg + 0.5 μg + 1 μg) (n = 10). (**b**) Current density versus membrane potential (from − 80 to 40 mV) shows that the conductance–current curve in *KCNQ2* WT + S247X + *KCNQ3* corresponds closely to the curve of the *KCNQ2* WT + *KCNQ3* WT cells. (**c**) Normalized current versus voltage for two conditions of *KCNQ2* WT + *KCNQ3* WT (1 μg + 1 μg) and *KCNQ2* WT + S247X + *KCNQ3* (0.5 μg + 0.5 μg + 1 μg). Currents in the heteromeric transfected S247X cells were approximately equal to those in the *KCNQ2* WT cells.

**Figure 4:**
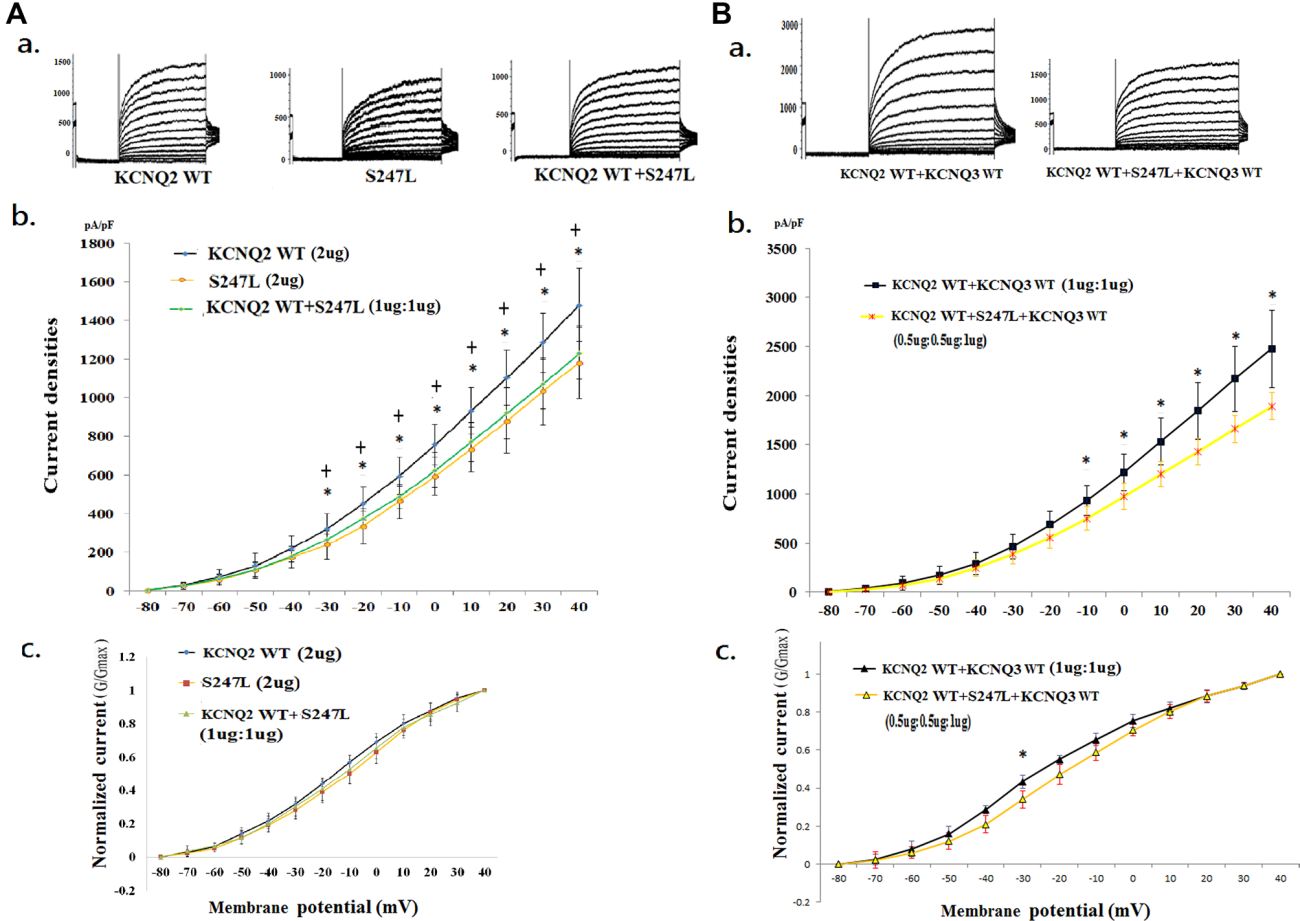
Analysis of the electrophysiological properties of HEK293 cells in KCNQ2 homomeric and heteromeric S247L channels. (**A**) (**a**) Representative current traces of *KCNQ2* WT (2 μg) (n = 20), S247L (2 μg) (n = 10), and *KCNQ2* WT + S247L (1 μg:1 μg) (n = 10). (**b**) Current density versus membrane potential (from − 80 to 40 mV) for *KCNQ2* WT, S247L, and *KCNQ2* WT + S247L. The current density in homomeric transfected variants demonstrated the lowest conductance–current curve in homomeric S247L. The conductance–current curves for heteromeric *KCNQ2* WT + S247L exhibited small increases and closely resembled the curve for homomeric S247L. *p < 0.05 for *KCNQ2* WT versus S247L; + *p* < 0.05 for *KCNQ2* WT versus *KCNQ2* WT + S247L. (**c**) Normalized currents (G/G_max_) versus voltage (from − 80 to 40 mV) for *KCNQ2* WT, S247L, and *KCNQ2* WT + S247L Cells transfected with homomeric S247L exhibited lower currents than did cells with the WT. (B) (a) Representative current traces of *KCNQ*2 WT + *KCNQ3* WT (1 μg + 1 μg) (n = 10) and *KCNQ2* WT + S247L + *KCNQ*3 (0.5 μg + 0.5 μg + 1 μg) (n = 10) are shown. (**b**) Current density versus membrane potential (from − 80 to 40 mV) for *KCNQ2* WT + *KCNQ3* WT and *KCNQ2* WT + S247L + *KCNQ3*. The conductance–current curve (from − 10 to 40 mV) in *KCNQ2* WT + S247L + *KCNQ3* differed from that of *KCNQ2* WT + *KCNQ3* WT cells (*p* < 0.05). *p < 0.05. (**c**) Normalized currents versus voltage in two conditions for *KCNQ2* WT + *KCNQ3* WT and *KCNQ2* WT + S247L + *KCNQ3*. *p < 0.05.

**Figure 5:**
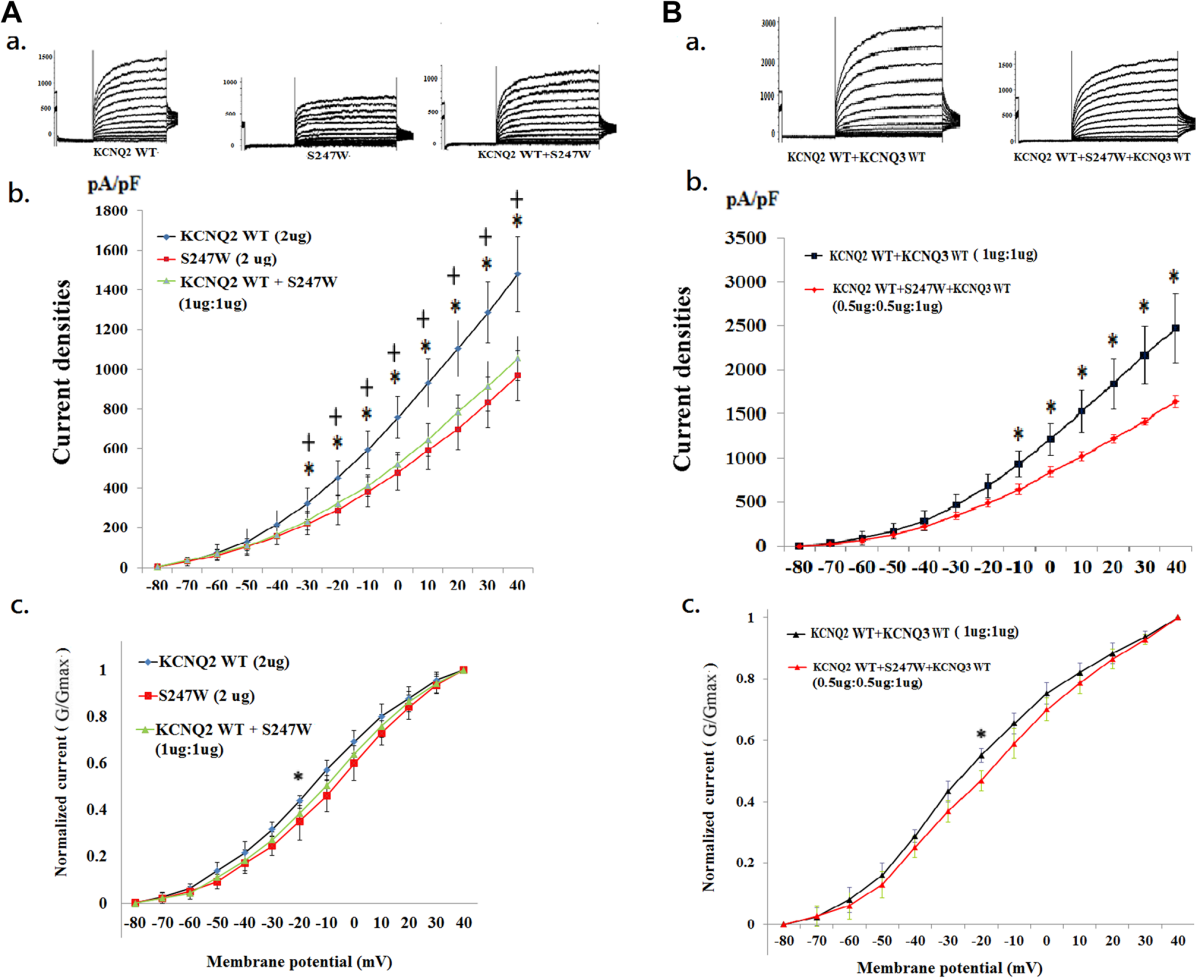
Analysis of the electrophysiological properties of HEK293 cells in KCNQ2 homomeric and heteromeric S247W channels. (**A**) (**a**) Representative current traces of *KCNQ2* WT (2 μg) (n = 20), S247W (2 μg) (n = 10), and *KCNQ2* WT + S247W (1 μg:1 μg) (n = 10). (**b**) Current density versus membrane potential (from − 80 to 40 mV) for *KCNQ2* WT, S247W, and *KCNQ2* WT + S247W. The current density in homomeric transfected variants demonstrated the lowest conductance–current curve in homomeric S247W from − 30 to 40 mV, and the conductance–current curve for *KCNQ2* WT + S247W exhibited small increases and was almost identical to the curve for S247W after the addition of *KCNQ2* WT*.* *p < 0.05 for *KCNQ2* WT versus S247W; + *p* < 0.05 for *KCNQ2* WT versus *KCNQ2* WT + S247W. (**c**) Normalized currents (G/G_max_) versus voltage (from − 80 to 40 mV) for *KCNQ2* WT, S247W, and *KCNQ2* WT + S247W. The cells transfected with homomeric S247X exhibited lower currents than did cells with the wild type. *p < 0.05 for *KCNQ2* WT versus S247W. (**B**) (**a**) Representative current traces of *KCNQ*2 WT + *KCNQ3* WT (1 μg + 1 μg) (n = 10) and *KCNQ2* WT + S247W + *KCNQ*3 (0.5 μg + 0.5 μg + 1 μg) (n = 10). (**b**) Current density versus membrane potential (from − 80 to 40 mV) for *KCNQ2* WT + *KCNQ3* WT and *KCNQ2* WT + S247W + *KCNQ3*. The conductance–current curve (from − 10 to 40 mV) in *KCNQ2* WT + S247W + *KCNQ3* differed that of the *KCNQ2* WT + *KCNQ3* WT cells (*p* < 0.05). *p < 0.05. (**c**) Normalized current versus voltage for *KCNQ2* WT + *KCNQ3* WT and *KCNQ2* WT + S247W + *KCNQ3*. *p < 0.05.

We analysed three mutations (i.e., S247L, S247W, and S247X) in which the cell M-current was affected after the transfection of homomeric or heteromeric variants. The homomeric channels were present in the S247X mutant (n = 10) (Fig. 3A, a), the S247L mutant (n = 10) (Fig. 4A, a), the S247W mutant (n = 10) (Fig. 5A, a), and the WT (n = 20). In the heteromeric channels, the mutations were transfected with heteromeric *KCNQ2* WT and mutants (1 μg: 1 μg) (*KCNQ2* WT + mutants [n = 10] [Figs. 3, 4, 5A, a]), heteromeric *KCNQ2* WT and variants, and *KCNQ3* WT [0.5 μg: 0.5 μg: 1 μg], with a DNA ratio mimicking the genetic balance (*KCNQ2* WT + mutants + *KCNQ3* WT [n = 10]) (Fig. 3, 4, 5B, a).

### Electrophysiological function in the S247X mutation of KCNQ2

The macroscopic currents were lowest in homomeric S247X and were less than 500 pA at + 40 mV in 7 out of 10 cells. The half activation potential (V_1/2_) was − 5.1 ± 3.6 mV; this value was right-shifted by 11.1 mV (*p* < 0.05) compared with the *KCNQ2* WT (− 16.2 ± 2.3 mV). The slope (K) was lower (3.1 ± 1.3; *p* < 0.005) compared with for the *KCNQ2* WT (9.4 ± 1.6) (Table 2). In HEK293 homomeric transfected variants, S247X cells expressed significantly lower currents than did the *KCNQ2* WT cells (p < 0.05) in each membrane potential (− 80 mV to 40 mV) (Fig. 3A, b). Moreover, the normalized current was significantly lower in homomeric S247X cells during episodes of conditional stimulation from − 30 to 10 mV (*p* < 0.05; Fig. 3A, c). After analysis of the nontransfected HEK293 cells, the current curve in nontransfected HEK293 cells was almost identical to the curve in the S247X cells (Table 2 and Supplementary Fig. S1), which indicated that homomeric S247X is nonfunctional. However, the conductance–current curve of S247X approached that of the WT after the addition of *KCNQ2* (Fig. 3A, b, c). The current density (pA/pF) in each membrane potential (− 80 mV to 40 mV) in homomeric and heteromeric transfected variants was lowest in S247X (Fig. 3A, b). The ratio of S247X to WT current density was 41.6% (Table 2). After transfection with heteromeric *KCNQ2* WT and variants and with *KCNQ3* WT (0.5 μg:0.5 μg:1 μg), with a DNA ratio mimicking the genetic balance, the conductance–current curve in S247X was similar to that of the WT (Fig. 3B, b, c).

**Table 2:** Current density and V_1/2_ in homomeric and heteromeric transfected HEK293 cell in the variants of S247X, S247L S247W, and P285T.

	N	V_1/2_ (mV) (mean ± SD)	K (slope) (mean ± SD)	Current densities (pA/pF) (mean ± SD)	Ratio of variants to WT current density (%)
*KCNQ2* WT^†^ (2 ug)	20	− 16.2 ± 2.3	9.4 ± 1.6	564.4 ± 49.9	100.0
Nontransfected HEK293 cell	5	− 5.2 ± 2.8*	2.6 ± 0.5**	213.5 ± 45.4**	37.8
S247L (2 ug)	10	− 12.1 ± 1.7*^&^	6.9 ± 1.9*^&^	419.2 ± 56.5*^&^	74.3
S247X (2 ug)	10	− 5.1 ± 3.6*	3.1 ± 1.3**	234.7 ± 35.8**	41.6
S247W (2 ug)	10	− 10.9 ± 3.0*^%^	5.7 ± 1.2**^%^	395.7 ± 40.0*^%^	70.1
P285T (2 ug)	10	− 13.8 ± 3.2^@^	7.9 ± 0.9*^@^	436.0 ± 55.0*^@^	77.2
*KCNQ2* WT + S247L (1ug:1 ug)	10	− 13.3 ± 0.7*^&^	7.8 ± 1.2*^&^	485.4 ± 24.5*^&^	86.0
*KCNQ2* WT + S247X (1ug:1 ug)	10	− 16.5 ± 1.0	9.1 ± 1.2	538.4 ± 31.4	95.4
*KCNQ2* WT + S247W (1ug:1 ug)	10	− 12.8 ± 0.8*^%^	6.3 ± 1.5**^%^	471.4 ± 33.4*^%^	83.5
*KCNQ2* WT + P285T (1ug:1 ug)	10	− 14.8 ± 2.1	8.8 ± 1.5	518.0 ± 24.5	91.8
*KCNQ2* WT + *KCNQ3* WT^#^ (1ug:1 ug)	10	− 20.8 ± 1.6	14.9 ± 3.0	813.9 ± 118.3	100.0
*KCNQ2* WT + S247L + *KCNQ3* WT (0.5 ug:0.5 ug:1 ug)	10	− 17.9 ± 0.9*^&^	12.1 ± 2.2	691.7 ± 51.4	85.0
*KCNQ2* WT + S247X + *KCNQ3* WT (0.5 ug:0.5 ug:1 ug)	10	− 20.37 ± 2.7	13.3 ± 1.8	788.9 ± 86.0	96.9
*KCNQ2* WT + S247W + *KCNQ3* WT(0.5 ug:0.5 ug:1 ug)	10	− 17.3 ± 1.4*^%^	10.7 ± 1.2**^%^	666.9 ± 41.2*^%^	81.9
*KCNQ2* WT + P285T + *KCNQ3* WT (0.5 ug:0.5 ug:1 ug)	9	− 18.8 ± 2.2	12.9 ± 1.8	705.2 ± 53.3	86.6

Data rounded off to the first decimal place.

WT, wild type; V is the test potential; V½, half-maximal activation voltage; SD, standard deviation.

The data were then fit to a Boltzmann distribution of the following form: *G/Gmax* = 1/(1 + *exp*[(*V* − *V*½)/*dx*]).

^†^The current densities in the homomeric transfected variants and heteromeric transfected *KCNQ2* WT + variants were compared with the current density in *KCNQ2* WT (2 ug) respectively.

^#^The current densities in the heteromeric *KCNQ2* WT + *KCNQ3* WT + variants were compared with the current density in *KCNQ2* WT + *KCNQ3* WT (1ug:1 ug) respectively.

*p < 0.05 compared with WT; **, *p* < 0.005; bold font indicates significantly different from WT.

^&^*p* < 0.05 in homomeric S247L versus homomeric S247X or heteromeric S247L versus heteromeric S247X respectively.

^%^*p* < 0.05 in homomeric S247W versus homomeric S247X or heteromeric S247W versus heteromeric S247X respectively.

^@^*p* < 0.05 in homomeric P285T versus homomeric S247X or heteromeric P285T versus heteromeric S247X respectively.

### Electrophysiological function in S247L and S247W of the KCNQ2 mutation

In HEK293 homomeric transfected variants, the V_1/2_ values were − 12.1 ± 1.7 mV in S247L and − 10.9 ± 3.0 mV in S247W; these values were both right-shifted compared with that of the *KCNQ2* WT (− 16.2 ± 2.3 mV; *p* < 0.05). The slope conductance was lower in S247L (6.9 ± 1.9; *p* < 0.05) and in S247W (5.7 ± 1.2; *p* < 0.005) than in the *KCNQ2* WT (9.4 ± 1.6) (Table 2). The current density ratios of S247L and S247W to WT were 74.3% and 70.1%, respectively (Table 2). The current density (pA/pF) in each membrane potential (− 80 mV to 40 mV) in homomeric transfected variants revealed the lowest current curve in homomeric S247L from − 30 to 40 mV (Fig. 4A, b) and in homomeric S247W from − 30 to + 40 mV (Fig. 5A, b). The homomeric transfected S247L and S247W cells expressed lower normalized currents than did the WT cells when they were individually transfected with the *KCNQ2* variants (Figs. 4A, c and 5A, c). In the heteromeric transfected HEK293 cells, the current densities were consistently lower in *KCNQ2* WT + S247L (86.0% of that for the *KCNQ2* WT) and in *KCNQ2* WT + S247W (83.5% of that for the *KCNQ2* WT) (Table 2; Figs. 4A, b and 5A, b). After the transfection with the heteromeric *KCNQ2* WT and variants and with the *KCNQ3* WT (0.5 μg:0.5 μg:1 μg), the V_1/2_ values were − 17.9 ± 0.9 mV in S247L and − 17.3 ± 1.4 in S247W; these values both differed (*p* < 0.05) from those for *KCNQ2* WT + *KCNQ3* WT (− 20.8 ± 1.6 mV). The slope conductance was consistently lower for *KCNQ2* WT + S247L + *KCNQ3* WT (0.5 μg:0.5 μg:1 μg) (12.1 ± 2.2) and *KCNQ2* WT + S247W + *KCNQ3* WT (0.5 μg: 0.5 μg: 1 μg) (10.7 ± 1.2; *p* < 0.005) than in *KCNQ2* WT + *KCNQ3* WT (1 μg:1 μg) (Table 2). In the heteromeric transfected HEK293 S247L and S247W cells, the current densities were consistently lower than those in the WT cells (Table 2; Figs. 4 and 5).

### Electrophysiological function in the novel P285T mutate on of KCNQ2

**Figure 6:**
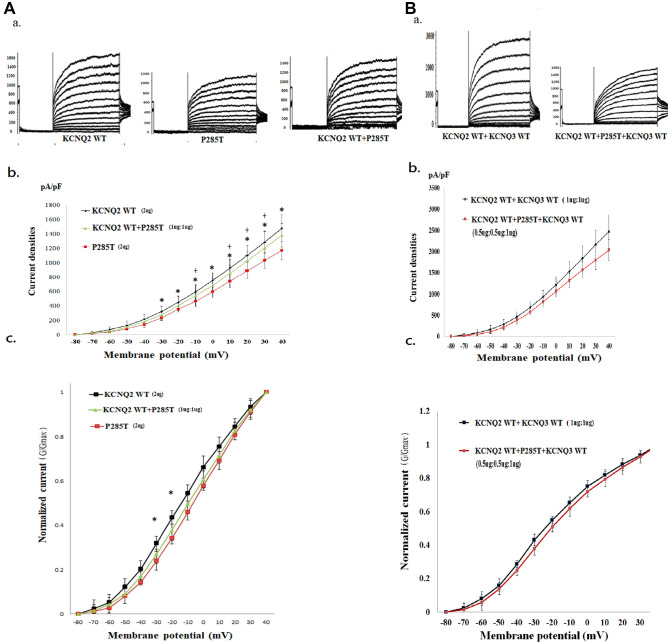
Analysis of electrophysiological properties of HEK293 cells in KCNQ2 homomeric and heteromeric P285T channels. (**A**) (**a**) Representative current traces of *KCNQ2* WT (2 μg) (n = 20), P285T (2 μg) (n = 10), and *KCNQ2* WT + P285T (1 μg:1 μg) (n = 10). (**b**) Current density versus membrane potential (from − 80 to + 40 mV) for *KCNQ2* WT, P285T, and *KCNQ2* WT + P285T. The current density in homomeric transfected P285T demonstrated the lowest conductance–current curve from − 30 to 40 mV. The conductance–current curves for *KCNQ2* WT + P285T exhibited smaller increases in the currents than did homomeric P285T*.* *p < 0.05 for *KCNQ2* WT versus P285T; + *p* < 0.05 for *KCNQ2* WT versus *KCNQ2* WT + P285T*.* (**c**) Normalized current (G/G_max_) versus voltage (from − 80 to 40 mV) for *KCNQ2* WT, P285T, and *KCNQ2* WT + P285T. The normalized currents were significantly lower in homomeric P285T (*p* < 0.05) during episodes of conditional stimulation from − 30 to − 20 mV. *p < 0.05 for *KCNQ2* WT versus P285T. (**B**) (**a**) Representative current traces of *KCNQ*2 WT + *KCNQ3* WT (1 μg + 1 μg) (n = 10) and *KCNQ2* WT + P285T + *KCNQ*3 (0.5 μg + 0.5 μg + 1 μg) (n = 9). (**b**) Current density versus membrane potential (from − 80 to 40 mV) for *KCNQ2* WT + *KCNQ3* WT and *KCNQ2* WT + P285T + *KCNQ3*. The conductance–current curve for *KCNQ2* WT + P285T + *KCNQ3* was lower than that for *KCNQ2* WT + *KCNQ3* WT cells. (**c**) Normalized current versus voltage for *KCNQ2* WT + *KCNQ3* WT and *KCNQ2* WT + P285T + *KCNQ3*. After heteromeric *KCNQ2* WT + P285T + *KCNQ3* WT transfection into HEK293 cells, the currents remained low.

In HEK293 homomeric transfected variants, the V_1/2_ was − 13.8 ± 3.2 mV; this value was right-shifted. The slope (K) was lower (7.9 ± 0.9; *p* < 0.05) than for the *KCNQ2* WT (Table 2). The P285T-to-WT current density ratio was 77.2% (Table 2). P285T cells expressed significantly lower current densities (pA/pF) than did the *KCNQ2* WT cells (*p* < 0.05) from − 30 to + 40 mV (Fig. 6A, b). The normalized currents were significantly lower in homomeric P285T cells for episodes of conditional stimulation from − 30 to − 20 mV than in HEK293 cells (Fig. 6A, c). In heteromeric transfected *KCNQ2* WT cells and variants (1 μg:1 μg), the conductance–current curves for *KCNQ2* WT + P285T exhibited smaller increases in currents than did homomeric P285T (Fig. 6A, b; Table 2). After the transfection with the heteromeric *KCNQ2* WT and variants and with the *KCNQ3* WT (0.5 μg:0.5 μg:1 μg), the current amplitudes remained lower in the P285T cells than in the *KCNQ2* WT + *KCNQ3* WT cells (Fig. 6B, b; Table 2).

### Comparisons of electrophysiological properties of S247L, S247W, and S247X

The homomeric normalized currents were lowest in S247X, followed by S247W, S247L, and the *KCNQ2* WT. V_1/2_ was significantly right-shifted in homomeric S247X compared with S247L and S247W in homomeric channels (*p* < 0.05). The slope conductance was significantly lower (*p* < 0.05) in homomeric S247X than in S247L and S247W (Table 2). In the heteromeric transfected *KCNQ2* WT and variants (1 μg: 1 μg), the amplitudes were lowest in S247W cells (*p* < 0.05) at a conditional voltage of − 20 mV and in S247L cells. After transfection with the heteromeric *KCNQ2* WT and variants and with the *KCNQ3* WT (0.5 μg: 0.5 μg: 1 μg), the normalized currents were lowest for S247W cells at − 20 mV (*p* < 0.05) and for S247L cells at − 30 mV (*p* < 0.05). The conductance–current curve was lowest in the S247W cells.The conductance–current curves for S247W and S247L exhibited smaller increases after the addition of *KCNQ2* and *KCNQ3* than did those for S247X (Figs. 4 and 5). In heteromeric transfected *KCNQ2* WT + *KCNQ3* + mutants, the current density in each membranous potential (− 80 mV to 40 mV) was highest in S247X cells, followed by S247L and S247W cells. In the homomeric channel, S247X had the lowest voltage; in the heteromeric channel, S247W and S247L had the lowest voltages. S247X had the lowest homomeric transfected cell current, but no significant impairments were noted in the heteromeric transfected S247X variant compared with in the WT.

### Phenotypes and KCNQ2 protein expression on cell membranes

**Figure 7:**
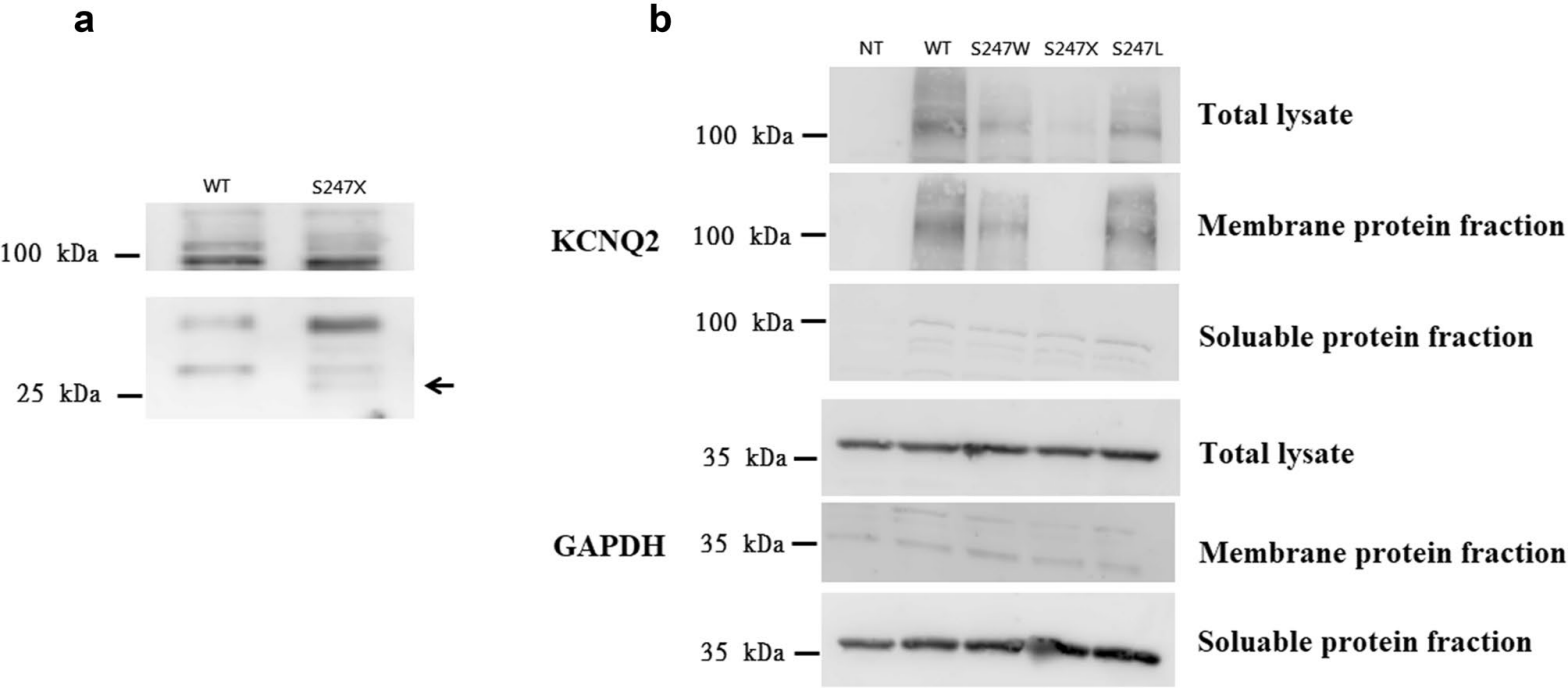
Western blotting demonstrated the protein expression in S247X, S247L, and S247W. (**a**) In contrast to the *KCNQ2* WT, KCNQ2 protein cell-membrane expression was not present in S247X (arrow). (**b**) Immunostaining with the anti-KCNQ2 antibody demonstrated the HEK293 cell-membrane expression of S247L, S247W, and S247X variants. Protein expressions were lower in S247L and S247W.

In the analysis of the KCNQ2 protein expression for variants (Fig. 7 and Supplementary Fig. S2–S4), KCNQ2 protein expression was absent in S247X (Fig. 7a). In S247L, S247W, and WT cells, the KCNQ2 proteins were expressed less than in the KCNQ2 WT (*p* < 0.05; Fig. 7b). The S247W and S247L phenotypes, however, were more prominent in patients with neonatal-onset EE. Protein expression was lowest in the S247X variant, but its phenotype was mild for the patient with BFNC. The genotypes of these mutation variants were uncorrelated with protein expression in cell membranes in those mutations.

### Neurodevelopmental outcomes related to the electrophysiological properties of S247L, S247W, S247X, and P285T mutations

All four mutations resulted in significantly lower conductance–current curves in homomeric channels compared with that in WT cells. The homomeric S247X cells demonstrated the lowest conductance–current curves. The heteromeric currents in the S247X cells were higher than the currents in the S247L, S247W, and P285T cells after transfection with *KCNQ2* + and *KCNQ2* + *KCNQ3* + mutants (Table 2). The neurodevelopmental outcomes were correlated with heteromeric Kv7.2 channels in these four mutations with *KCNQ2* neonatal-onset EE and *KCNQ2* BFNC.

## Discussion

We confirmed that heteromeric *KCNQ2* and *KCNQ3* channels compensated for the Kv7.2 functional current changes when concurrently transfected with heteromeric *KCNQ3*, *KCNQ2*, and mutants. Functional current changes were more severe in S247X in homomerically transfected cells; however, the current changes were less severe in S247X cells, but not in S247W or S247L cells, when concurrently heteromerically transfected with *KCNQ3* and *KCNQ2* mutants. The heteromeric functional current impairment in S247W was the largest among S247X, S247L and S247W. Tryptophan protein (W) is bulky and might narrow the KCNQ2 protein pore domain based on the molecular model. The prediction of the molecular model is compatible with the results of research on electrophysiological properties. Several studies have claimed that homomeric current change is common in *KCNQ2* neonatal-onset EE^28,29,36,40^. In contrast, we found that heteromeric functional current changes are correlated with long-term neurodevelopmental outcomes. The finding increases our understanding of poor cognition of children with *KCNQ2* neonatal-onset EE despite the seizure had been got remission. That could increase our understanding to manage affected patients.

The M-current changes in mutations varied depending on the locations of mutations and their consequences. The consequences might be partially or completely related to the clinical phenotype and neurodevelopmental outcomes. However, the factors contributing to long-term neurodevelopmental outcomes are multiple and complex, including genotype, genetic mosaicism, modified genes, duration of seizures, and environmental factors^6–8,37^. Homomeric current changes in *KCNQ2* mutations were common in *KCNQ2* mutations, suggesting loss of the ability to regulate neuronal firing^40^. However, in this study, the homomeric current changes were not necessarily associated with later neurodevelopmental outcomes. This study revealed that the “rescue” effects after the addition of *KCNQ2* and *KCNQ3* were more closely correlated with the neurodevelopmental outcomes. This is supported in *KCNQ2* encephalopathy observed in one study with A294V^29^ and in the present study. We hypothesized that S247X with nonsense-mediated decay^46–48^ activates compensation with more KCNQ2 and KCNQ3 expression in vivo and presents the BFNC phenotype. By contrast, S247L and S247W were presumed not to initialize the mechanism due to mild or negative effects on protein expression. This is also supported by the results of a mouse study^47^; the *KCNQ2* gene was truncated in mice, and quantitative polymerase chain reaction (PCR) revealed an absence of *KCNQ2* mRNA in ganglia from *KCNQ2* (-/-). Indeed, mRNA levels in *KCNQ3* and *KCNQ5* for these truncated genes were approximately doubled^49^.

Although the S247X variant causes less protein assembly on the cell membrane, its phenotype is milder than are the S247L and S247W phenotypes. The variant that causes the most severe phenotype is not associated with protein expression on HEK293 cell membranes. The macroscopic currents were present in S247X cells but were extremely low, with the curve of these currents closely resembling that of nontransfected HEK293 cells. Our study revealed that the S247X currents could be markedly improved in the heteromeric channels that were predicted to be associated with more favourable outcomes for the phenotype associated with BFNCs than for the S247L and S247W phenotypes associated with neonatal-onset EE. Among the *KCNQ2* mutations, most of the truncation, nonsense, and splicing mutations resulted in favourable BFNC phenotypes according to The Human Gene Mutation Database (https://www.hgmd.cf.ac.uk/ac/index.php), even for mutations located in voltage-sensing domains or in pore regions. The mechanism involved is obscure. KCNQ3 is already expressed in most of the brain structure during the 3 final months of the gestational period, and no major variations apply after birth^50^. In the mouse study^49^, the *KCNQ2* gene was truncated in the mice, and quantitative PCR showed an absence of *KCNQ2* mRNA in ganglia from KCNQ2 (–/–) but a 100–120% increase in KCNQ3 and KCNQ5. In nonsense mutations such as S247X, although the entire C-terminus was absent, the heteromeric channel including KCNQ3 could play a role that renders the Kv7.2 functional. The hypothesis is verified in clinical phenotypes for which seizures improved after the age of 1 month. We predict that the S247X nonsense mutation caused a lower expression of the KCNQ2 protein due to nonsense-mediated mRNA decay^46–48^, which activated hetero-KCNQ2 and KCNQ3 functional compensation. However, a decrease in the expression of the KCNQ2 protein in S247L and S247W was not predicted. One study found that the protein expression on cell membranes due to missense mutations in voltage sensor and pore domain did not decrease^28^. We hypothesized that the missense mutations cannot cause hetero-KCNQ2 and KCNQ3 functional compensation as patients age.

More than 100 different *KCNQ2* genotypes have been described in the literature thus far, but the phenotypes cannot be accurately predicted by the genotypes. Mutations in the voltage-sensing domains (VSDs) and pore regions might affect the channel gating function and contribute to severe phenotypes; however, mutations on the C-terminal (M546V^7^, R547W^51^, R553Q^52^, R553W^6^, and R553L^6^) also cause neonatal-onset EE, but the mechanism is not clear. The probable mechanism was some C-terminal mutations have been reported^38,53^ to interact with CaM and to affect Kv7.2 channels that cause severe neonatal-onset EE. Some *KCNQ2* mutations found in the S4 and pore regions have a more severe dominant-negative effect and lead to neonatal-onset EE, and *KCNQ2* mutation variants in the S4 domain can affect channel gating and increase the threshold for channel activation^38,53^, but the mutations in the S4 domain do not significantly impair surface protein expression^53^.

The genotype–phenotype relationship is complex. Haploinsufficiency is caused by the loss of function of a single *KCNQ2* allele, including nonsense, splice, or frameshift mutations of *KCNQ2*, which is the most common genotype of familial *KCNQ2*-BFNC. The nonsense, splice, and frameshift variants cause nonfunctional or partially functional KCNQ2 proteins and BFNCs, and they contribute to better outcomes than do missense mutations in the critical functional domain of KCNQ2 proteins. The probable in vivo mechanisms are compensated by hetero*-*KCNQ2 and KCNQ3 subunits or modified genes. The hypothesis can be supported by a reduction in functional impairment after the addition of *KCNQ2* and *KCNQ3*. We recommend that the amount of KCNQ2 protein cell-membrane expression and its association with the phenotype be further investigated. The mechanisms in the *KCNQ2* variant that underlie neonatal seizures in the pore and voltage-sensing domains, and variants in the C-terminal should be different. Determining what they are should lead to better treatments and outcomes.

This study has some limitations. A functional study in vitro using HEK293 cells with a potassium channel might contribute to a bias in currents. When recorded the HEK293 cells without transfected *KCNQ2*, the currents closely resembled those for homomeric S247X, which indicated that homomeric S247X was not functional in the present study. That is compatible with S247X by nonsense-mediated mRNA decay^46–48^ and removing all C-terminal domain for tetramerisation of functional channels, is supposed to be nonfunctional. HEK 293 cells have been widely used in cell biology and the gene expression in HEK 293 cells is like that of the neuron. Their non-expression of potassium ion channels makes them a good model for patch-clamp recordings because there are minor interfering currents.

## Conclusions

Our findings support the notion that homomeric current changes are common in *KCNQ2* neonatal-onset EE and *KCNQ2* BFNC; however, heteromeric functional current changes are correlated with long-term neurodevelopmental outcomes.

## Supplementary information

Supplementary Information
