# Supplementary material for: Heteromeric Kv7.2 current changes caused by loss-of-function of KCNQ2 mutations are correlated with long-term neurodevelopmental outcomes
Source: Sci Rep. 2020 Aug 7;10:13375. doi: 10.1038/s41598-020-70212-w (PMC7415140; doi:10.1038/s41598-020-70212-w)
Supplement: Supplementary file 1 — Supplementary Information [file 41598_2020_70212_MOESM1_ESM.docx]

**Heteromeric Kv7.2 current changes caused by loss-of-function of *KCNQ2* mutations are correlated with long-term neurodevelopmental outcomes**

***Inn-Chi Lee, Jiann-Jou Yang, Swee-Hee Wong, Ying-Ming Liou, Shuan-Yow Li***

**
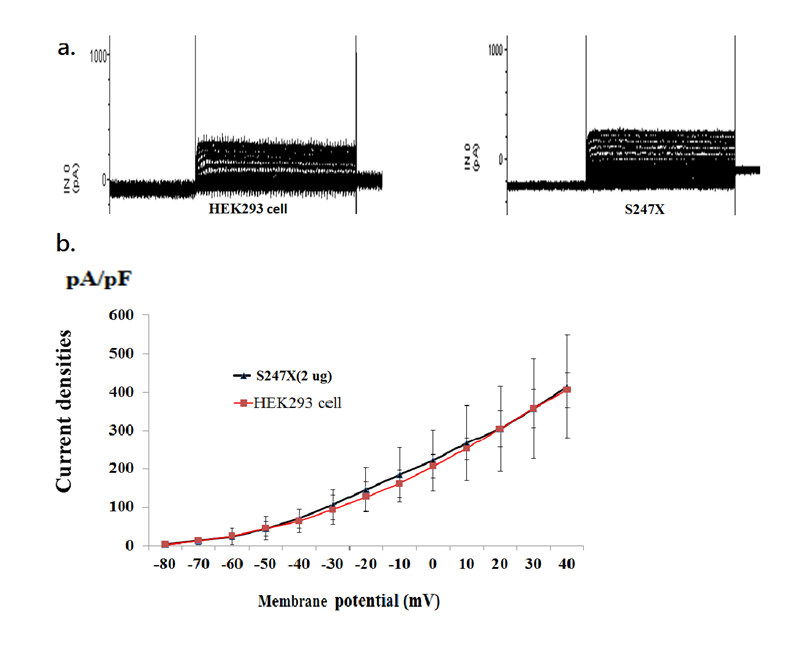
**

**Supplementary Figure** S1. Analysis of the electrophysiological properties of nontransfected HEK293 cells (n = 5) and S247X (2 μg) channels. (a) Representative current traces of nontransfected HEK293 cells and S247X. (b) Current density versus membrane potential (from −80 to 40 mV) for nontransfected HEK293 cells and S247X. The current curve in nontransfected HEK293 cells was almost identical to that in S247X cells.

**
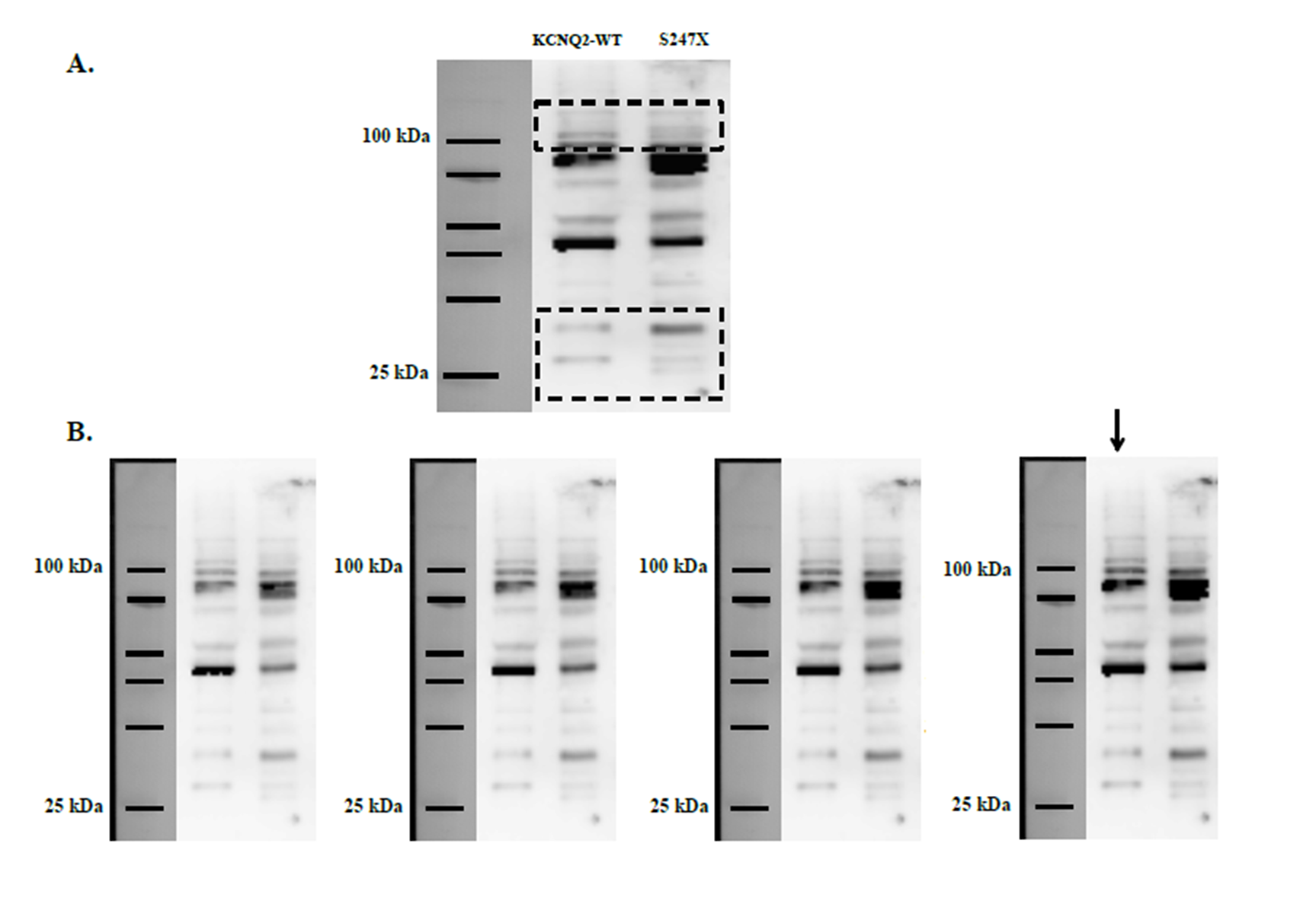
**

**Supplementary Figure S2. (A)** Original gels before cropping of dashed-line regions depicted in Figure 10a. **(B)** Original gels include multiple exposure images of the same gel. Arrow indicates the selected original image.

**
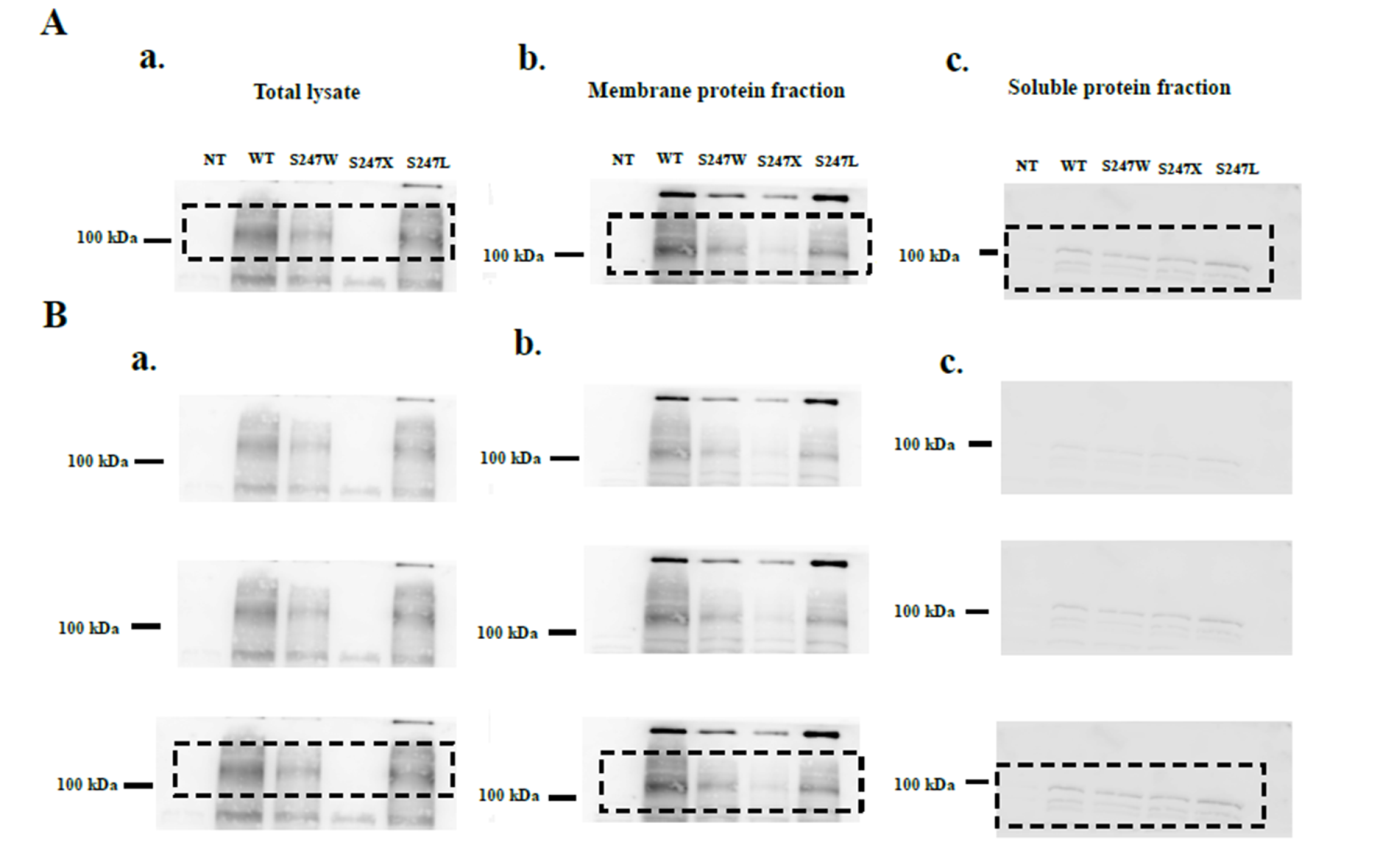
**

**Supplementary Figure S3. (A)** Original gels before cropping of dashed-line regions depicted in Figure 10b; (a) total lysate, (b) membrane protein fraction, and (c) soluble protein fraction. **(B)** Original gels include multiple exposure images of the same gel; (a) total lysate, (b) membrane protein fraction, and (c) soluble protein fraction. Arrows indicates the selected original images. Dashed line represents the cropped region.

**
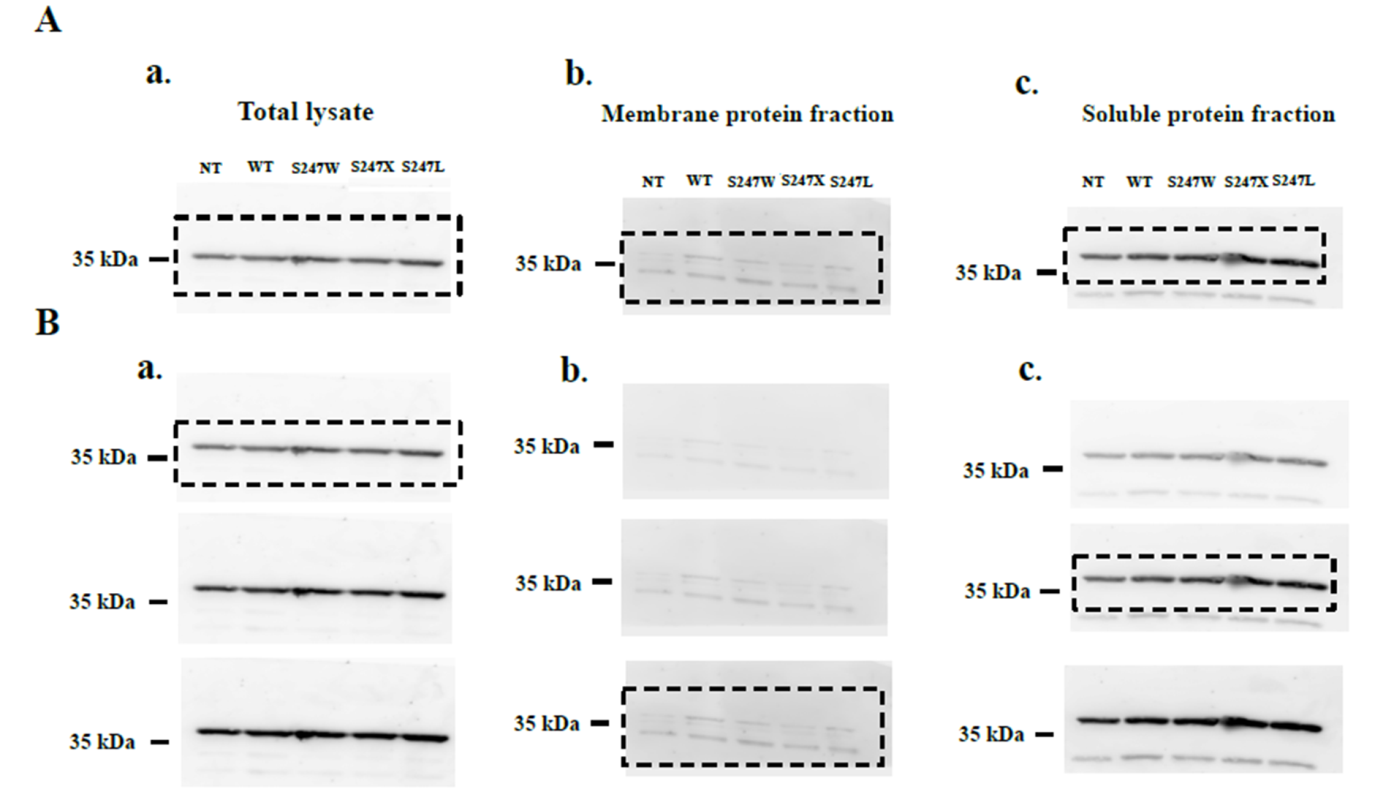
**

**Supplementary Figure S4. (A)** Original gels before cropping of dashed-line regions depicted in Figure 10b, GAPDH; (a) total lysate, (b) membrane protein fraction, and (c) soluble protein fraction. **(B)** Original gels include multiple exposure images of the same gel in Figure 10b, GAPDH; (a) total lysate, (b) membrane protein fraction, and (c) soluble protein fraction. Arrows indicates the selected original images. Dashed line represents the cropped region.
